# Letter to editor regarding ‘Insight into the history and trends of surgical simulation training in education: a bibliometric analysis’ – reflections for the current day

**DOI:** 10.1097/JS9.0000000000000849

**Published:** 2024-07-12

**Authors:** H. Cook, D. Zargaran, A. Mosahebi

**Affiliations:** a Department of Plastic Surgery, Royal Free Hospital; b Division of Surgery and Interventional Sciences, University College London, UK

*Dear Editor*,

We read with interest your article entitled “Insight into the history and trends of surgical simulation training in education: a bibliometric analysis”^1^. The rapid development in simulation training in recent years and incorporation of technology is both encouraging and exciting. The demonstrable impact of simulation techniques such as virtual reality (VR) are likely to be impactful on future medical education.

The authors note that simulation now comprises a significant part of clinical training and is resource-intensive. With this in mind, we would like to further discuss the role of VR augmented reality to further contribute to medical education in an increasingly resource-pressured medical education environment. VR has the potential to be both reproducible and personalised. The authors discuss the advantages of haptic and real-time feedback with regards to VR training. We feel it is important to also consider other ways that VR education can be tailored to the learner – learning can be performed in a location and at a time of choice and exercises can be repeated until a learner feels comfortable. A 2023 trial by Peters *et al*.^2^ in this journal demonstrated a VR course teaching suturing to be comparable to a tutor-led course and significantly better than an e-learning course in both qualitative and quantitative measures. The authors raise an important point that with much of surgical skills training, repetition, and familiarity are important to success, and therefore, providing learners with the maximal opportunity to develop and practice skills is likely to improve skill acquisition the most^2^.

Within certain specialties, such as plastic surgery, realistic, and cost-effective simulation has been difficult to achieve thus far. Human cadaveric models pose difficulties with ethical considerations and high costs. Animal cadaveric specimens can lack adequate similarity to patients^3^. Simulation via VR can decrease costs significantly for procedural training in skills such as microsurgery. Aesthetic training performed virtually allows for real-time correction and ‘undo’ functions which allows learners to assess the success of their practice^3^.

The advent of artificial intelligence (AI) also creates a potential space for learners to have tailor-made virtual learning. A lack of individualised feedback has been a major drawback of virtual learning environments to date, however, the use of AI to create automated assessment may go some way to negate this. Machine learning has been suggested as a way to not only evaluate performance but also predict areas of trainee weakness and areas of focus^3^.

This learning approach also has benefits for patients and the wider health system. By enabling learners to make mistakes safely and not enacting skills on patients without adequate experience, patients are protected from avoidable mistakes made in the novice period^4^. From a health system perspective, education can be delivered in a smaller space, with less need for personnel, thus freeing up faculty^4^. Education can also be delivered more broadly to individuals unable to access it otherwise, for example, in less economically developed countries, leading to more equitable surgical education and delivery of care^4^.

## Ethical approval

Not applicable.

## Consent

Not applicable.

## Source of funding

Not applicable.

## Author contribution

H.C.: concept and writing; D.Z.: writing; A.M.: concept and manuscript checking.

## Conflicts of interest disclosure

Not applicable.

## Research registration unique identifying number (UIN)

Not applicable.

## Guarantor

Hannah Cook.

## Data availability statement

Due to the nature of this submission as a correspondence, no new data have been collected in its creation or writing.

## Provenance and peer review

Not applicable.
